# Letter from Paris

**Published:** 1867-12

**Authors:** 


### CORRESPONDENCE.

---

*Letter from Paris.*

PARIS, *October* 10, 1867.

MESSRS. EDITORS:

THE remarkable effects of Bromide of Potassium as a revulsive and anæsthetic agent now renders the study of its physiological action a matter of considerable importance.

In the *Journal des Connaissances Medicales* we find a paper on the subject by Drs. Eulenburg and Guttmann, who have effected experiments with it on rabbits as well as frogs. The sub-cutaneous injection of from two to four grammes of bromide of potassium produces on rabbits a perturbation on the action of the heart, accompanied by diminished sensibility and weaker voluntary action: it kills them in the course of from 10 to 40 minutes with the symptoms of paralysis of the heart.

This paralysis is not retarded by continuing respiration artificially by means of tracheotomy. If instead of injection, the same quantity of the substance be injected under the form of a solution in four parts of water, it will kill with the same symptoms, corroding the mucous membrane of the stomach.

Smaller doses are seldom mortal, the symptoms produced are ephemeral and sometimes preceded by slight shivers. Similar effects to those described are produced on frogs with doses of from six to nine hundredths of a gramme. Hence it appears that bromide of potassium exercises a special action on the heart; but does not operate in a direct manner on the peripheric nerves or on the muscles.

But in all these effects potassium seems to be the real agent, quite independently of bromine. The latter, even injected pure in much larger doses than those above employed, exercises no particular action on the heart or on the nervous system, and does not kill the animal. Frogs will resist the inhalation of bromic emanations. Nor has bromide of sodium any effect similar to those of bromide of potassium, for it kills very slowly, merely producing great general weakness.

The adulteration of Bromide of Potassium by Iodide of Potassium has become such a serious question in a therapeutic point of view, that it is a matter of great interest to find some easy way of ascertaining it. Several ways have been proposed, but none of a very practical kind and possessing at the same time a sufficient degree of exactness.

A paper by M. Amédie Blacher, in the *Journal des Connaissances Medicales*, is on this account deserving of notice.

Mr. Blacher starts from the following facts:—1. Bromide of potassium yields a white precipitate with salts of lead; iodide of potassium a yellow one.

2. Pyroligneous acid dissolves iodide of lead, especially at the temperature of ebullition.

3. It also dissolves bromide of lead.

4. Bromide of lead is more soluble in pyroligneous acid than the iodide of the same metal. The two latter observations are due to Mr. Blacher himself.

Now when at the common temperature, pyroligneous acid is added to a precipitate of bromide of lead mixed with iodide of the same, the latter is detected by the yellow spots which almost instantly appear in the midst of the white mass.

Moreover the iodide may be separated from the bromide by cooling their solution in a liquid composed of one part of pyroligneous acid and twelve of water for in this case the iodide is precipitated while the bromide remains in the liquid. The former may then be collected and weighed. Mr. Blacher's plan of operations may now be easily understood.

Suppose we have some bromide of potassium which we have reason to suspect of being adulterated with iodide.

We transform the bromide in question into that of lead by adding to its solution in water an excess of liquid sub-acetate of lead, a substance which will equally decompose the iodide of potassium.

On examining the precipitate found, if it be white, there is no iodide in it, if on the contrary, it be tinged with orange, we shall have prima facie evidence of the presence of iodide. In order to determine the quantity of the latter, the precipitate must be washed and dried, then dissolved in the pyroligneous liquid above described, and this solution being put into cold water, the iodide will precipitate and the bromide will remain in the liquid.

The Exposition is nearly finished and I have scarcely had time to visit it. What I saw in the dental department, is scarcely worthy of notice outside of the fine specimens of of artificial teeth manufactured in America. A very thorough account will be published I believe at the expense of the government, when it appears I will send it to you.

D'Oyley-Evans.

